# Children’s and adolescents’ perceptions of Discovery Colleges in Germany: low awareness but high acceptability

**DOI:** 10.3389/fpsyt.2026.1892661

**Published:** 2026-09-04

**Authors:** Jared Omundo, Manuel Föcker, Simon Alexander Stiehl, Shiblu Miah, Michael Schulz, Orkan Okan

**Affiliations:** 1 TUM Health Literacy Unit, Department of Health and Sport Sciences, TUM School of Medicine and Health, Technical University of Munich, Munich, Germany; 2 Recovery College Gütersloh (RC GT-OWL), LWL-Klinikums Gütersloh, Gütersloh, Germany; 3 LWL Klinikum Gütersloh, Gütersloh, Germany; 4 Pflegerische Abteilungsleitung Allgemeinpsychiatrie, Honorar-Professur an der Fachhochschule der Diakonie, Bielefeld, Germany; 5 Department of Child and Adolescent Psychiatry, University Hospital Münster, Münster, Germany; 6 LWL-Universitätsklinik Hamm, Hamm, Germany; 7 Child and Adolescent Psychiatry, Psychotherapy, and Psychosomatics, Ruhr University-Bochum, Gütersloh, Germany; 8 Medizinische Fakultät, Medizinische Hochschule Brandenburg Theodor Fontane, Neuruppin, Germany; 9 Hochschule Osnabrück – University of Applied Science, Osnabrück, Germany; 10 Recovery College Osnabrück (RCO), Osnabrück, Germany; 11 Discovery College Practice, Youth Mental Health, RECOIL – Flourish in Education, Luton, United Kingdom

**Keywords:** adolescents, Discovery Colleges, Germany, mental health literacy, Recovery Colleges, youth mental health

## Abstract

**Background:**

Mental health challenges among children and adolescents are increasing globally and in Germany, highlighting the need for accessible, youth-centred, and prevention-oriented approaches. Discovery Colleges (DCs) are recovery-oriented, co-produced educational models that promote mental health literacy, empowerment, and peer learning within non-clinical settings. Although Discovery Colleges have been implemented internationally, little is known about how young people in Germany perceive this approach. This study explored children’s and adolescents’ awareness, perceived relevance, willingness to engage, and preferences regarding Discovery Colleges within the German context.

**Methods:**

A cross-sectional mixed-methods online survey was conducted between January and April 2026 among children and adolescents aged 10–18 years. Following predefined data-cleaning procedures, responses with less than 70% questionnaire completion were excluded, resulting in a final analytic sample of 227 participants. The survey assessed familiarity with Discovery Colleges, perceived relevance following a standardised description of the model, willingness to participate, previous experiences with mental health support, sociodemographic characteristics, and open-ended questions exploring perceived facilitators and barriers to participation. Quantitative data were analysed descriptively, while qualitative responses were analysed using reflexive thematic analysis.

**Results:**

Awareness of Discovery Colleges was low, with only 5.9% of participants reporting prior familiarity with the concept. Following a brief standardised description, 64.8% perceived Discovery Colleges as relevant for youth mental health support, and 82.4% expressed willingness to participate if such a programme were available locally. Qualitative findings identified four themes influencing engagement: (1) visibility and youth-centred outreach, (2) social connectedness and peer engagement, (3) accessibility and low-threshold participation, and (4) safe, non-clinical environments.

**Conclusions:**

Despite limited prior awareness, many children and adolescents perceived Discovery Colleges as relevant and reported a willingness to engage with this type of youth-oriented mental health approach. These findings suggest that increasing awareness and ensuring accessible, youth-friendly implementation may support the future introduction of Discovery Colleges within the German mental health system. Given the exploratory design and non-probability sample, the findings should be interpreted cautiously and require confirmation through future implementation and evaluation studies.

## Highlights

- What is known:
  Youth mental health needs are increasing globally, yet a substantial proportion of young people do not access appropriate support. Existing services often fail to engage young people because of structural barriers, stigma, and limited alignment with their preferences.
- What is new:
  Although awareness of Discovery Colleges was very low, many adolescents perceived the approach as relevant and expressed a willingness to participate after receiving a brief description. These findings suggest that limited awareness does not necessarily correspond to low acceptability of youth-oriented, recovery-oriented educational approaches.
- Clinical implications:
  Discovery Colleges may represent a potentially valuable youth-centred and preventive complement to existing mental health services in Germany. Increasing awareness and ensuring accessible, youth-friendly implementation may support future engagement with recovery-oriented mental health initiatives.

## 1 Introduction

Mental health difficulties commonly emerge during childhood and adolescence and constitute a major contributor to the global burden of disease among young people, with significant long-term implications for educational attainment, social functioning, and occupational participation (1, 2). Despite the availability of effective interventions, a substantial proportion of children and adolescents do not receive appropriate mental health support. Persistent barriers include limited mental health literacy, stigma, structural inequalities, long waiting times, and difficulties navigating fragmented systems of care (3–6). These challenges have intensified calls for innovative approaches that complement traditional treatment-oriented services by promoting prevention, early intervention, participation, and recovery within community and educational settings (2, 7, 8). In response, increasing attention has been directed towards recovery-oriented, participatory, and educational approaches that extend mental health support beyond conventional clinical care. Rather than focussing exclusively on symptom reduction, recovery-oriented approaches emphasise empowerment, co-production, lived experience, agency, and meaningful participation as central mechanisms for promoting wellbeing and recovery (9, 10). Within this broader movement, educational and non-clinical interventions have emerged as accessible, low-threshold opportunities to strengthen mental health literacy, reduce stigma, foster social connectedness, and encourage earlier engagement with mental health support (11–13).

**Figure 1:**
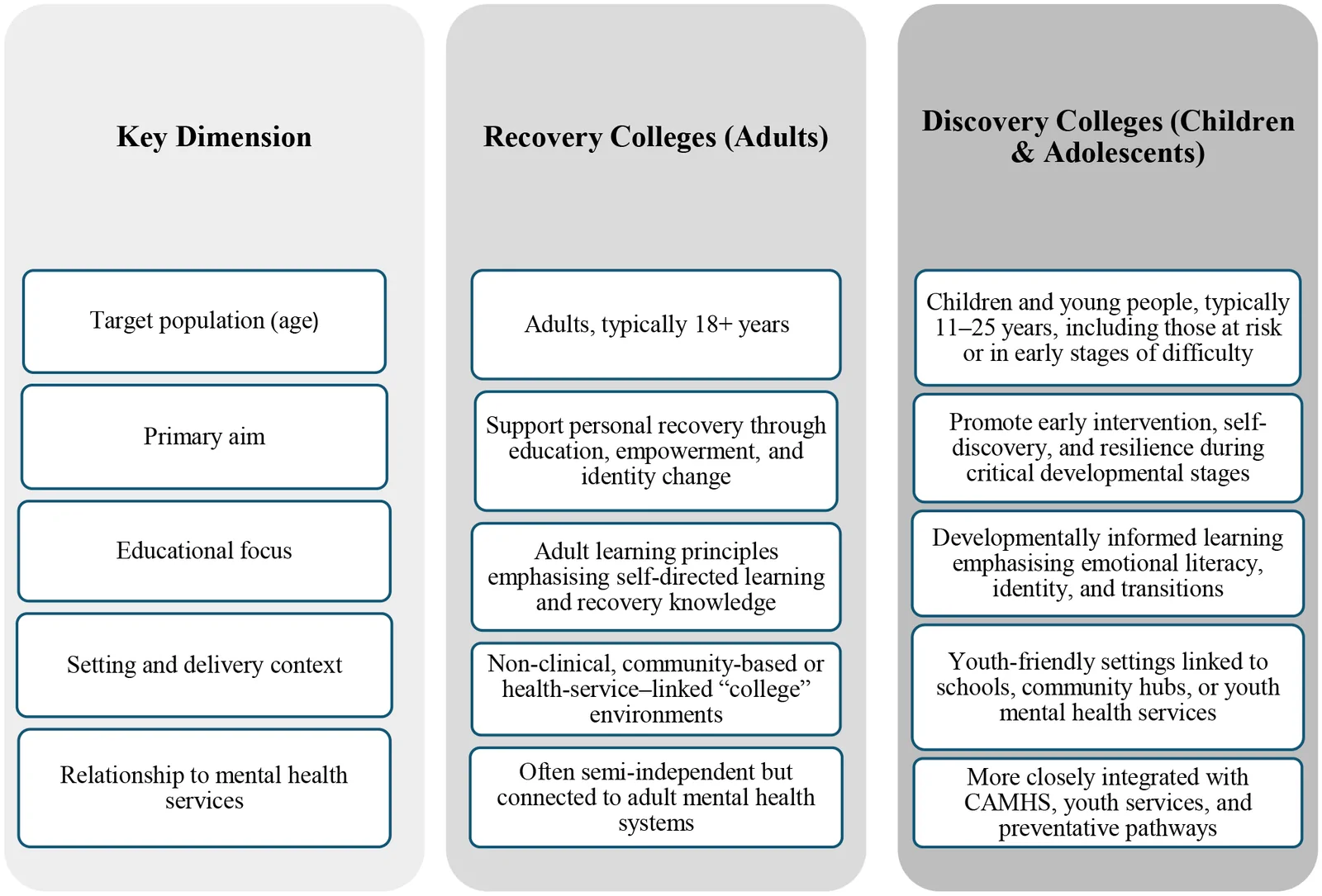
Comparison of Recovery Colleges and Discovery Colleges. Adapted from Omundo et al. (2026), licensed under the Creative Commons Attribution 4.0 International License (CC BY 4.0; https://creativecommons.org/licenses/by/4.0/).

Discovery Colleges (DCs) represent one emerging example of this recovery-oriented educational approach. Originally developed in Australia, Discovery Colleges are co-produced, recovery-oriented learning environments specifically designed for children, adolescents and young adults (14, 15). They bring together young people, mental health professionals, family members and individuals with lived experience to collaboratively design and deliver educational courses on mental health, wellbeing, resilience, and recovery (15–17). Rather than positioning participants as patients or service users, Discovery Colleges conceptualise them as learners and active contributors to their own wellbeing. Through peer learning, co-production, and experiential knowledge, they aim to create non-clinical learning environments that promote empowerment, self-efficacy, social connectedness, and meaningful engagement with mental health support. Courses are typically delivered as structured workshops or short educational programmes within schools, community organisations, universities, and youth mental health services. Common topics include emotional wellbeing, stress management, resilience, mental health literacy, communication, coping strategies, relationships, and self-care. Sessions are interactive and dialogical, integrating peer exchange, reflective exercises, and collaborative learning processes. Teaching is delivered jointly by mental health professionals and individuals with lived experience, recognising both forms of expertise as equally valuable. This collaborative educational model reflects the broader philosophy of recovery-oriented practice while simultaneously integrating prevention, health promotion, and youth participation within accessible community settings. Discovery Colleges are conceptually informed by the international Recovery College movement but should not be understood simply as Recovery Colleges for younger populations. Recovery Colleges were originally developed within adult mental health services as recovery-oriented educational environments that promote personal recovery through co-production, peer support, and adult learning principles (18–20). Discovery Colleges build upon these foundational principles while extending them to address the developmental, educational, and social realities of childhood and adolescence. Consequently, they place greater emphasis on prevention, mental health literacy, emotional development, family involvement, educational transitions, and community engagement than traditional adult Recovery Colleges. As illustrated in **Figure 1**, Discovery Colleges therefore represent a developmentally responsive reinterpretation of recovery-oriented educational innovation rather than a direct adaptation of the adult Recovery College model.

Although Discovery Colleges retain core recovery-oriented principles, including co-production, peer learning, experiential knowledge, and empowerment, they are situated within contemporary youth mental health systems where prevention and early intervention are central priorities (21). The philosophy underpinning Discovery Colleges is broadly consistent with recovery frameworks such as CHIME, which conceptualises recovery through connectedness, hope, identity, meaning, and empowerment (9). However, Discovery Colleges operationalise these principles within developmentally appropriate educational settings that seek not only to support personal recovery but also to strengthen mental health literacy, resilience, participation, and wellbeing among children and adolescents. Emerging international evidence suggests that Discovery Colleges may improve mental health literacy, empowerment, confidence, social connectedness, and wellbeing while providing accessible and non-stigmatising pathways into mental health support (15, 17). However, the current evidence base remains comparatively limited and is largely concentrated in Australia and the United Kingdom. Recent reviews have highlighted the need for implementation research examining how Discovery Colleges can be introduced, adapted, and embedded within different cultural, organisational, and healthcare contexts (21). This need is particularly relevant in Germany, where Discovery Colleges have not yet been systematically implemented. Although a range of initiatives seek to promote youth mental health and mental health literacy, existing approaches remain heterogeneous, fragmented, and predominantly project-based rather than embedded within coordinated recovery-oriented systems of care (22, 23). However, little is currently known about how children and adolescents themselves perceive Discovery Colleges or whether they consider such approaches relevant, acceptable, and engaging within the German context. Understanding these perspectives is essential because young people’s acceptance and willingness to participate represent important prerequisites for the successful implementation of recovery-oriented educational innovations.

### 1.1 Study aim and research questions

The present study aims to explore the perceptions of children and adolescents in Germany regarding Discovery Colleges as a recovery-oriented educational innovation within youth mental health systems. Specifically, the study examines young people’s awareness, perceived relevance, acceptability, willingness to engage and perceived facilitators and barriers to participation in a context where Discovery Colleges have not yet been systematically implemented.

The study addresses the following research questions:

1. What level of awareness and familiarity do children and adolescents in Germany have regarding Discovery Colleges?
2. How do children and adolescents perceive the relevance and acceptability of Discovery Colleges?
3. How willing are young people to engage with Discovery College approaches?
4. What facilitators and barriers do children and adolescents perceive regarding potential participation in Discovery Colleges?

By generating empirical evidence on young people’s perceptions of Discovery Colleges, this study seeks to inform the context-sensitive adaptation and future implementation of recovery-oriented educational innovations within the German youth mental health system. The findings are intended to contribute to the emerging evidence base on Discovery Colleges and to identify areas for future implementation and conceptual research.

## 2 Methods

### 2.1 Study design and procedure

A cross-sectional mixed-methods study was conducted between January and April 2026 using an anonymous online survey to explore children’s and adolescents’ awareness, perceptions, and willingness to engage with Discovery Colleges (DCs) as a recovery-oriented educational approach within youth mental health systems. Given that Discovery Colleges have not yet been systematically implemented in Germany, an exploratory design was considered appropriate to generate preliminary empirical evidence regarding young people’s awareness, perceived relevance, acceptability, and perceived facilitators and barriers to participation. The study targeted children and adolescents aged 10–18 years residing in Germany, with data collection primarily conducted in North Rhine-Westphalia and Bavaria. Participants were recruited through schools, youth organisations, and social media platforms. To maximise reach and access to diverse youth populations, a non-probability sampling strategy combining convenience and snowball sampling was employed. Participation was voluntary. Eligible participants were children and adolescents aged 10–18 years residing in Germany who provided assent to participate and, where required, written parental or legal guardian consent. Participants were excluded if they were younger than 10 years, older than 18 years, unable to complete the questionnaire because of severe cognitive impairment, or did not provide the required consent or assent. Prior to participation, respondents received an age-appropriate information sheet outlining the study aims, procedures, participant rights, and data protection measures. Written informed consent was obtained from parents or legal guardians, alongside assent from all participants. The survey required approximately 8–10 minutes to complete.

As Discovery Colleges are currently largely unknown within the German context, participants were provided with a brief standardised description of the model prior to answering perception-related questions to ensure a shared baseline understanding of the concept. The questionnaire assessed:

- sociodemographic characteristics;
- previous experiences of mental health support;
- awareness of Discovery Colleges;
- perceived relevance and acceptability;
- willingness to engage with Discovery Colleges and;
- perceived facilitators and barriers to participation through open-ended questions.

Quantitative items primarily consisted of categorical and Likert-type response formats. Open-ended questions were included to capture participants’ views regarding factors that may facilitate or hinder participation in Discovery Colleges and considerations relevant to their future implementation in Germany. Upon completion of the survey, participants received a short debriefing together with information about available youth mental health support services. All data were collected anonymously.

### 2.2 Measures

#### 2.2.1 Sociodemographic characteristics

Sociodemographic variables included age, gender, federal state of residence, school type, and previous experience with mental health support. School type was assessed according to the German education system, which comprises several educational pathways that differ in academic orientation and qualification levels. Response options included Grundschule (primary school), Hauptschule (lower secondary school), Realschule (intermediate secondary school), Gymnasium (academic secondary school leading to the *Abitur* and university entrance), Gesamtschule (comprehensive secondary school), and other educational settings. For the statistical analyses, school types were grouped into low, medium, and high educational tracks, reflecting the degree of academic orientation within the German education system and facilitating interpretation for an international readership.

#### 2.2.2 Self-reported need for mental health support

Subjective need for mental health support was assessed using a dichotomous item asking participants whether they had ever needed help because they were not feeling well (“yes”/”no”). The measure was intended to capture perceived need for support independent of formal diagnosis or service utilisation.

#### 2.2.3 Awareness, acceptability, and willingness to engage

Participants were asked whether they had prior familiarity with Discovery Colleges (“yes”/”no”). Following presentation of the standardised description, participants assessed the perceived relevance of Discovery Colleges for youth mental health promotion (“yes”/”no”) and indicated their willingness to participate using a three-category response format (“yes, definitely”, “maybe”, “no”).

#### 2.2.4 Open-ended responses

Participants were invited to provide free-text responses regarding factors that might motivate young people to attend Discovery Colleges and recommendations concerning how such programmes should be designed and implemented within the German context.

### 2.3 Sampling and data collection

The survey instrument was developed based on existing literature concerning recovery-oriented educational models, Recovery Colleges, and Discovery Colleges. Prior to the main data collection, the questionnaire was piloted with 50 children and adolescents to assess clarity, usability, and developmental appropriateness. Feedback from participants and parents resulted in minor revisions to wording, structure, and item comprehension, thereby supporting face validity and age-appropriateness. Data collection was conducted using the SoSci Survey platform and could be accessed through both a web link and QR code across multiple digital devices. To maximise participation, respondents were encouraged to share the survey link within their networks as part of the snowball recruitment strategy. As an incentive, participants could voluntarily enter a prize draw to win one of twenty €10 vouchers allocated through a computer-generated randomisation procedure.

### 2.4 Data analysis

Quantitative analyses were conducted using IBM SPSS Statistics (Version 29). Prior to analysis, the dataset underwent screening and data cleaning procedures. To ensure data quality while maintaining adequate sample retention, a completion threshold was applied. Completion rates were calculated as the proportion of completed questionnaire items relative to the total number of questionnaire items. Cases with less than 70% completion were excluded according to a predefined completion criterion. The application of completion thresholds is consistent with established recommendations for survey-based and eHealth research (24). Additional data quality checks included inspection of questionnaire completion times and response patterns to identify potentially invalid questionnaires.

Given the exploratory aims of the study, quantitative analyses consisted of descriptive statistics, including frequencies and percentages, to summarise participant characteristics and the main study variables. These analyses were used to describe participants’ awareness of Discovery Colleges, perceived relevance and acceptability, willingness to engage, previous experiences of mental health support, and preferences regarding Discovery College programmes. Qualitative responses were analysed using reflexive thematic analysis following Braun and Clarke (25, 26). An inductive analytic approach was employed, whereby codes and themes were generated iteratively from the data rather than being informed by predefined theoretical frameworks. Coding and theme development involved ongoing reflective discussions within the research team to enhance reflexivity, transparency, and the credibility of the interpretive process. Consistent with reflexive thematic analysis, these discussions were intended to support interpretation rather than establish inter-rater reliability.

### 2.5 Ethical considerations

Ethical approval for the study was obtained from the Ethics Committee of the Technical University of Munich (TUM; reference number: 2025-57-NM-BA). The study was conducted in accordance with the Declaration of Helsinki and relevant national ethical guidelines. Participation was voluntary. Written informed consent procedures were implemented in accordance with ethical requirements for research involving minors, including assent from participants and parental or guardian consent where applicable. No personally identifiable information was collected, and all responses were anonymous. Participants were informed that participation was voluntary and that they could discontinue participation at any time prior to survey submission. All data were handled in accordance with applicable data protection regulations.

## 3 Results

### 3.1 Sample characteristics

A total of 624 survey responses were initially recorded. Following data cleaning procedures, questionnaires with more than 30% missing responses were excluded. The final analytic sample comprised 227 participants who had completed at least 70% of the questionnaire. Age data were available for 220 participants. Most participants were aged 15–18 years (62.7%), while 37.3% were aged 10–14 years. Gender information was available for 208 participants, of whom 58.2% identified as female, 39.9% as male, and 1.9% as gender diverse. Geographic distribution based on postal code regions indicated a strong concentration of participants from southern Germany, particularly Bavaria, with smaller proportions from other federal regions including North Rhine-Westphalia, Baden-Württemberg, and additional northern and Eastern German states. Most respondents were currently attending school (89.9%), with smaller proportions enrolled in university education (4.3%), vocational training (1.4%), or other educational or occupational settings (4.3%). Participants represented a range of educational pathways within the German school system. For descriptive purposes, school types were grouped into low, medium, and high educational tracks. Most participants attended schools within the medium (56.1%) or high (33.8%) educational tracks, whereas 10.1% were enrolled in the low educational track. Regarding previous mental health experiences, approximately half of the participants (49.3%) reported that they had previously felt the need for mental health support, while 50.7% reported no previous perceived need. Participant characteristics are summarised in **Table 1**.

**Table 1:** Sample characteristics (N = 227).

Variable	Category	N	% (valid)
Age	10–14 years	82	37.3
	15–18 years	138	62.7
Gender	Female	121	58.2
	Male	83	39.9
	Diverse	4	1.9
Geographic Region (Federal State)	Bavaria (Bayern)	177	78.0
	North Rhine-Westphalia	23	10.0
	Baden-Württemberg	7	3.0
	Other Federal States*	20	9.0
Current Status	School Student	186	89.9
	Vocational Training	3	1.4
	University Student	9	4.3
	Other	9	4.3
Formal Education	Low	20	10.1
	Medium	111	56.1
	High	67	33.8
Previous Experience with Mental Health Support	Yes	100	49.3
	No	103	50.7

The study was designed to recruit participants from Bavaria and North Rhine-Westphalia; however, additional responses were obtained from other federal states* (Berlin/Brandenburg, Saxony, and Northern Germany).

### 3.2 Awareness, perceived relevance, and willingness to participate

Awareness of Discovery Colleges was limited among participants. Of the 185 participants who provided valid responses, only 5.9% reported they had previously heard of Discovery College, whereas 94.1% indicated no prior familiarity with the concept. Participants who were unfamiliar with Discovery Colleges subsequently received a brief description of the concept and were asked whether they perceived the approach as relevant for youth mental health support. Among valid responses (n = 193), 64.8% indicated that they considered Discovery Colleges relevant following the explanation, whereas 34.7% did not. Participants were also asked whether they would be willing to participate in a Discovery College if such a programme were available in their local community. Responses were subsequently recoded into a binary willingness variable reflecting openness versus unwillingness to participate. Among valid responses (n = 204), the majority of participants (82.4%) expressed at least some willingness to participate, whereas 17.6% indicated that they would not be willing to participate.

### 3.3 Perceived facilitators and barriers to participation

Analysis of the open-ended responses identified four themes describing factors that participants perceived as important for engaging with Discovery Colleges. These themes related to visibility and communication, opportunities for peer interaction, accessibility and programme delivery, and the characteristics of the learning environment (**Table 2**).

**Table 2:** Overview of themes identified from the qualitative analysis.

Theme	Description	Illustrative participant responses
Visibility and Youth-Centred Outreach	Participants emphasised the importance of increasing awareness of Discovery Colleges through youth-oriented communication strategies, including social media, schools, community organisations, and trusted role models.	“Effective advertising through social media, schools and places where young people spend time…” (P31); “Introducing Discovery Colleges in schools or providing clear explanations through videos on social media.” (P04); “Good social media advertising where young people can see the real people who will actually be there.” (P34); “Promotion through role models, influencers, clubs, the media and schools.” (P12).
Social Connectedness and Peer Engagement	Participants valued opportunities to connect with peers, build friendships, share experiences, and participate alongside others in supportive group environments.	“Meet people with similar interests and make new friends.” (P62); “It would be easier if friends from school or leisure activities also came along.” (P87); “Taking part as a group is important because it is much harder to leave your comfort zone alone.” (P56); “Meet new people, be creative, relax and simply feel free.” (P73).
Accessibility and Flexible Participation	Participants highlighted the importance of practical accessibility, including free participation, flexible scheduling, convenient locations, diverse activities, and autonomy over participation.	“Making the programme free of charge, offering flexible schedules and choosing a central location.” (P11); “Different activities for different interests, flexible attendance and suitable opening times.” (P22); “Close to where young people live, with food, drinks and a wide variety of activities.” (P46); “Young people should decide for themselves which activities they want to take part in.” (P27).
Safe and Youth-Friendly Learning Environments	Participants described a preference for engaging, creative, welcoming, and psychologically safe learning environments that differed from school or traditional mental health services.	“Creative courses with a wide range of activities such as archery, photography, creative expression and sports.” (P16); “A programme that does not feel like school, allows young people to try new things and gives them freedom.” (P104); “A genuine safe space where people are taken seriously.” (P91); “A trusted person, a comfortable environment, no pressure to speak, and confidence that personal privacy will be respected.” (P52).

### 3.4 Theme 1: visibility and youth-centred outreach

Participants frequently identified communication and promotion as important facilitators of engagement with Discovery Colleges. Many emphasised that information should be disseminated through communication channels commonly used by young people, particularly social media, schools, and community organisations. One participant suggested that Discovery Colleges should use “effective advertising through social media, schools and places where young people spend time, such as clubs, organisations or music schools. Posters and flyers could be displayed there, and information stands at public events would also help. Once young people have positive experiences, word of mouth would encourage others to attend” (P31). Another participant recommended “introducing Discovery Colleges in schools or providing clear explanations through videos on social media” (P04), while another highlighted the importance of “good social media advertising where young people can see the real people who will actually be there” (P34). Participants also suggested involving trusted messengers, including “role models, influencers, clubs, the media and schools,” to increase awareness of Discovery Colleges (P12).

### 3.5 Theme 2: social connectedness and peer engagement

Participants frequently described opportunities for social interaction and peer connection as important facilitators of participation. Several respondents emphasised the value of meeting peers with similar interests or experiences. One participant explained that “many young people would probably attend if they realised they could try new things that are different from school, meet people with similar interests and make new friends. It also helps if they can see that it might benefit them later in life while still being fun and giving them freedom” (P62). Others highlighted that participation would feel easier “if friends from school or leisure activities also came along” (P87), while another respondent noted that “taking part as a group is important because it is often much harder to leave your comfort zone alone, whereas being with others gives you a sense of security” (P56). Participants also described Discovery Colleges as places where they could “meet new people, be creative, relax and simply feel free” (P73).

### 3.6 Theme 3: accessibility and flexible participation

Participants frequently identified practical and organisational factors as important facilitators of participation. Many emphasised that programmes should be free, easily accessible, and flexible enough to fit around young people’s daily lives. One participant recommended “making the programme free of charge, offering flexible schedules and choosing a central location in the city” (P11). Another suggested that Discovery Colleges should provide “different activities for different interests, flexible attendance and suitable opening times” (P22), while another explained that participation would be easier if “it is close to where young people live, offers food and drinks, and provides a wide variety of activities for both introverted and extroverted young people” (P46). Participants also highlighted the importance of having autonomy over their participation, with one respondent stating that young people should be able to “decide for themselves which activities they want to take part in” (P27), ensuring that participation remains voluntary and self-directed.

### 3.7 Theme 4: safe and youth-friendly learning environments

Participants frequently described the importance of engaging, creative, and supportive learning environments. Many expressed a preference for programmes that were enjoyable, flexible, and clearly different from school or traditional mental health services. One participant suggested that Discovery Colleges should offer “creative courses with a wide range of activities such as archery, photography, different forms of creative expression and sports” (P16). Another explained that “many young people would participate if the programme did not feel like school, allowed them to try new things, gave them freedom, and helped them discover what they enjoy” (P104). Participants also emphasised the importance of a welcoming and psychologically safe atmosphere. One respondent described Discovery Colleges as “a genuine safe space where people are taken seriously and where attending is accepted rather than something to feel embarrassed about” (P91). Another participant highlighted the importance of “a trusted person, a comfortable environment, no pressure to speak, and confidence that personal privacy will be respected” (P52).

## 4 Discussion

### 4.1 Principal findings

The present study explored how children and adolescents in Germany perceive Discovery Colleges as a recovery-oriented educational approach within youth mental health systems. Specifically, it examined young people’s awareness of Discovery Colleges, their perceived relevance, willingness to participate, and the factors they considered important for engaging with such programmes. To our knowledge, this is among the first empirical studies to investigate perceptions of Discovery Colleges within the German context. Four principal findings emerged. First, awareness of Discovery Colleges was very limited among participants. Second, following a brief description of the concept, many participants perceived Discovery Colleges as a relevant approach for supporting youth mental health. Third, most participants expressed a willingness to participate should such programmes become available. Finally, participants identified several factors that they considered important for encouraging engagement, including youth-centred communication, opportunities for peer interaction, accessible programme delivery, and safe, youth-friendly learning environments. The following sections discuss these findings in relation to existing literature on Discovery Colleges, Recovery Colleges, youth mental health literacy, and recovery-oriented practice, and consider their implications for future research and implementation.

### 4.2 Awareness, perceived relevance and willingness to participate

One of the principal findings of this study was that awareness of Discovery Colleges among children and adolescents was very limited. However, after receiving a brief description of the concept, many participants perceived Discovery Colleges as relevant for supporting youth mental health, and most expressed a willingness to participate should such programmes become available. This descriptive pattern suggests that unfamiliarity with Discovery Colleges does not necessarily imply a lack of interest in recovery-oriented educational approaches. Given that Discovery Colleges have not yet been systematically introduced within Germany, low awareness was expected and is likely to reflect limited exposure rather than established attitudes towards the model. Although comparable studies examining awareness of Discovery Colleges are currently lacking, these findings are broadly consistent with evidence indicating that adolescents often have limited mental health literacy prior to targeted educational interventions (27, 28). They also align with previous research showing that educational interventions can increase understanding and improve attitudes towards mental health, although changes in help-seeking behaviour and stigma are generally more modest (29, 30). The findings should, however, be interpreted cautiously. Participants evaluated Discovery Colleges after receiving only a brief description of the concept rather than through direct experience of participation. Consequently, the study assessed perceived relevance and anticipated willingness to participate rather than actual engagement or programme effectiveness. Future implementation studies are therefore needed to examine whether these positive perceptions are maintained when Discovery Colleges are introduced in practice.

### 4.3 Perceived facilitators of engagement

The qualitative findings provide further insight into the programme characteristics that participants considered important for encouraging engagement with Discovery Colleges. Young people consistently highlighted youth-oriented communication, opportunities for peer interaction, accessible programme delivery, and welcoming learning environments. These qualitative findings provide additional context for interpreting the quantitative results by illustrating how participants would prefer Discovery Colleges to be introduced and delivered within the German context. The emphasis on communication through social media, schools, and trusted organisations is consistent with research demonstrating that adolescents primarily access health information through digital media and educational settings (27). Similarly, participants’ preference for peer interaction reflects broader evidence highlighting the importance of social relationships, shared experiences, and peer support in youth mental health promotion (6, 31). Participants also emphasised practical considerations, including free participation, flexible scheduling, and convenient locations, suggesting that accessibility is an important consideration when designing youth-oriented programmes. Participants further described a preference for creative, welcoming, and non-judgemental learning environments that differed from traditional school or clinical settings. Similar characteristics have previously been reported in Recovery Colleges and Discovery Colleges internationally, where educational approaches, peer learning, and supportive environments are considered important features of recovery-oriented practice (21). Although the present study did not examine programme effectiveness, these findings provide preliminary insight into the characteristics that young people perceive as important when engaging with novel mental health initiatives.

### 4.4 Implications for practice and policy

Although exploratory, the present findings have several practical implications for organisations considering the introduction of Discovery Colleges within Germany. First, the very low level of prior awareness suggests that implementation efforts should include clear communication and public engagement strategies to introduce the concept to young people, families, schools, and community organisations. Second, participants consistently emphasised the importance of delivering Discovery Colleges through accessible, youth-friendly, and non-stigmatising environments that complement existing educational and community settings. Finally, participants’ preferences for peer interaction, flexible programme delivery, and creative learning activities suggest that these characteristics should be considered during future adaptation of Discovery Colleges within the German context. These implications should be interpreted as practical considerations informed by participants’ perceptions rather than as evidence of programme effectiveness or implementation success. Future pilot studies and implementation research are needed to evaluate how Discovery Colleges can be adapted, delivered, and integrated within the German youth mental health system and to examine their impact on mental health literacy, wellbeing, help-seeking, and recovery-oriented outcomes.

### 4.5 Strengths and limitations

To our knowledge, it represents one of the first empirical investigations of children’s and adolescents’ perceptions of Discovery Colleges within Germany and, more broadly, German-speaking contexts, thereby addressing an important gap in the emerging literature on recovery-oriented educational approaches for young people. By examining an internationally established model within a new national context, the study provides preliminary evidence regarding the perceived relevance and acceptability of Discovery Colleges and contributes to discussions concerning the adaptation of youth-oriented mental health innovations across healthcare and educational systems. The mixed-methods design enabled the integration of quantitative and qualitative findings, providing complementary insights into both the prevalence of perceptions and the factors that participants considered important for engagement.

Several limitations should also be acknowledged. First, the study employed a non-probability sampling strategy and recruited participants primarily from Bavaria and North Rhine-Westphalia. Consequently, the findings cannot be considered representative of all children and adolescents in Germany. Although the analytic sample was adequate for an exploratory mixed-methods study, the number of valid responses was modest after data cleaning, and the results should therefore be interpreted with appropriate caution. Participation was voluntary and conducted online, which may have introduced selection bias by preferentially including young people with internet access or an existing interest in mental health topics (32, 33). In addition, adolescents from socioeconomically disadvantaged backgrounds or lower educational pathways may have been underrepresented, despite often experiencing greater barriers to mental health support (5, 34). Second, the study relied on self-reported perceptions elicited after participants received a brief standardised description of Discovery Colleges. Consequently, the findings reflect anticipated relevance and willingness to participate rather than actual experience with or participation in Discovery Colleges. The cross-sectional design also precludes conclusions regarding future engagement, implementation, or programme effectiveness. Third, although Discovery Colleges are founded on principles of co-production and youth participation, young people were not involved in the design of the present study. The research questions, survey instrument, and study procedures were developed by the research team rather than through a co-design process with young people. This represents an important limitation, as involving young people in the development of the study may have strengthened the relevance of the survey content and ensured that the dimensions of acceptability reflected their priorities, language, and lived experiences more closely. Finally, the qualitative findings were derived from open-ended survey responses, which generally provided less depth than qualitative interviews or focus groups. Although these responses yielded valuable insights into participants’ perceptions, they should be regarded as exploratory and descriptive rather than a comprehensive account of young people’s experiences and preferences.

### 4.6 Future research

The present findings provide an initial understanding of how children and adolescents perceive Discovery Colleges within the German context and identify several areas for future investigation. First, pilot implementation studies are needed to examine the feasibility, acceptability, and sustainability of Discovery Colleges across different educational, community, and healthcare settings. Such studies should evaluate how Discovery Colleges can be adapted to the German youth mental health system while maintaining the core principles of recovery-oriented education. Second, longitudinal and implementation research is required to examine whether young people’s initial willingness to participate translates into actual engagement and whether participation contributes to improvements in mental health literacy, wellbeing, recovery-oriented outcomes, and help-seeking behaviours. Such studies would provide important evidence regarding the effectiveness of Discovery Colleges within routine practice. Third, future research should adopt co-produced approaches that actively involve children and adolescents in the design, implementation, and evaluation of Discovery Colleges as well as the research itself. Involving young people throughout the research process would ensure that future interventions and evaluation strategies better reflect their priorities, preferences, and lived experiences. Finally, future implementation research may explore the processes through which awareness, perceived relevance, organisational readiness, and stakeholder engagement influence the successful introduction and sustainability of Discovery Colleges. Such process-oriented research should employ appropriate implementation science methodologies capable of empirically examining these relationships rather than inferring them from exploratory cross-sectional data.

## 5 Conclusion

This study provides one of the first empirical insights into how children and adolescents in Germany perceive Discovery Colleges as a recovery-oriented educational approach within youth mental health systems. Although prior awareness of Discovery Colleges was limited, many participants perceived the approach as relevant following a brief introduction and expressed a willingness to participate should such programmes become available. Participants also identified several factors that they considered important for engagement, including youth-oriented communication, opportunities for peer interaction, accessible programme delivery, and safe, welcoming learning environments. Taken together, these findings provide preliminary evidence that Discovery Colleges may be perceived positively by young people within the German context and identify practical considerations that may inform future adaptation and implementation efforts. However, given the exploratory nature of the study, the use of a non-probability sample, and the absence of direct experience with Discovery Colleges among participants, the findings should be interpreted cautiously. Future pilot implementation studies, longitudinal evaluations, and co-produced research are now needed to determine how Discovery Colleges can be successfully adapted, implemented, and evaluated within the German youth mental health system.

## Data Availability

The raw data supporting the conclusions of this article will be made available by the authors, without undue reservation.
